# Social Determinants of Chronic Prostatitis/Chronic Pelvic Pain Syndrome Related Lifestyle and Behaviors among Urban Men in China: A Case-Control Study

**DOI:** 10.1155/2016/1687623

**Published:** 2016-08-04

**Authors:** Yan Wang, Chen Chen, Changcai Zhu, Liang Chen, Qingrong Han, Huarong Ye

**Affiliations:** ^1^Department of Preventive Medicine, School of Public Health, Wuhan University of Science and Technology, 2 Huangjia Lake West Road, Hongshan District, Wuhan, Hubei 430065, China; ^2^Yiling Hospital, 31 East Lake Avenue, Yiling District, Yichang, Hubei 443100, China; ^3^China Resources & WISCO General Hospital, 209 Metallurgy Road, Qingshan District, Wuhan, Hubei 430080, China

## Abstract

*Purpose.* In order to find key risk factors of chronic prostatitis/chronic pelvic pain syndrome (CP/CPPS) among urban men in China, an age-matched case-control study was performed from September 2012 to May 2013 in Yichang, Hubei Province, China.* Methodology.* A total of 279 patients and 558 controls were recruited in this study. Data were collected by a self-administered questionnaire, including demographics, diet and lifestyle, psychological status, and a physical exam. Conditional logistic regression model was used to analyze collected data.* Results.* Chemical factors exposure, night shift, severity of mood, and poor self-health cognition were entered into the regression model, and result displayed that these four factors had odds ratios of 1.929 (95% CI, 1.321–2.819), 1.456 (95% CI, 1.087–1.949), 1.619 (95% CI, 1.280–2.046), and 1.304 (95% CI, 1.094–1.555), respectively, which suggested that these four factors could significantly affect CP/CPPS.* Conclusion.* These results suggest that many factors affect CP/CPPS, including biological, social, and psychological factors.

## 1. Introduction

Chronic prostatitis/chronic pelvic pain syndrome (CP/CPPS) is a chronic pain disorder, which is characterized by the presence of noninfectious pelvic or perineal pain lasting longer than 3 months. The International Prostatitis Collaborative Network of the National Institutes of Health (IPCN-NIH) has provided detailed criteria for diagnosing CP/CPPS [1, 2]. According to various epidemiological studies using different methodologies, the prevalence of CP/CPPS varies from approximately 8% to 20% worldwide [3–6]. A population-based survey estimated that the prevalence of CP/CPPS-like symptoms in China is 4.5% [4].

Although there have been many basic and clinical research studies, the exact etiology, pathophysiology, and mechanism of CP/CPPS remain indeterminate. This syndrome is currently considered to be a multifactorial medical condition and requires a multimodal treatment approach [7]. New diagnostic/therapeutic criteria targeted to the urinary, psychosocial, organ-specific, infection, neurological/systemic, and tenderness (UPOINT) system were developed by Shoskes et al. in 2009 to classify patients suffering with CP/CPPS and, more importantly, to direct appropriate therapy [8]. Multimodal therapy based on the UPOINT phenotype system greatly improves the symptoms of CP/CPPS [9, 10].

Currently, CP syndromes represent an important healthcare problem worldwide [11]. Furthermore, many studies have suggested that CP places a large financial burden on patients and society. Chronic prostatitis increases healthcare expenditures directly and indirectly (e.g., unemployment). The average total costs (direct and indirect) for 3 months of CP treatment is USD 1099 per person for resource consumption, with an expected annual total cost per person of USD 4397 [12]. In China, treatment for CP is relatively costly (USD 1151 or 8059 CNY per person) [13].

A cross-sectional study about CP/CPPS patients has been done previously; it was reported that there were many potential factors that might have an influence on CP/CPPS, including smoking, drinking tea, sedentariness, overstress, economic pressure, and self-health cognition [14, 15]. However, as a cross-sectional study they could not give more information about risk factors and affected degree. Therefore, based on the results of the cross-sectional study several factors were selected and a case-control study was conducted for further study of risk factors of CP/CPPS.

## 2. Materials and Methods

### 2.1. Study Setting and Target Population

This case-control study was conducted in Yiling District, Yichang, Hubei Province, China. The study included one administrative district, one economic development zone, eight towns, and three townships. With a total area of 3424 square kilometers and a population of 546,500, this district is the most populous administrative region of Yichang. Most of the residents are drivers or laborers. The target populations of this study are composed of male patients diagnosed with CP/CPPS for the first time in Yiling Hospital and healthy men without CP/CPPS symptoms in Yiling District. Data were collected from 279 patients with CP/CPPS between September 2012 and May 2013. Five hundred and fifty-eight males matched by age (1 : 2) without CP/CPPS symptoms were enrolled into the control group.

### 2.2. Selection Criteria for Cases and Controls

Diagnosis of patients with CP/CPPS was based on the IPCN-NIH consensus [1] following a critical medical interview, a digital rectal examination, a prostate secretion examination after prostate massage, and urinalysis. Patients who suffered from pyuria and other genitourinary symptoms that may be associated with benign prostatic hyperplasia, chronic bacterial prostatitis, acute prostatitis, or genitourinary diseases other than CP/CPPS were excluded from the study to minimize the measurement bias. The control subjects were healthy men without CP/CPPS symptoms who participated in a physical examination in Yiling Hospital. People who had been diagnosed with benign prostatic hyperplasia, chronic bacterial prostatitis, acute prostatitis, or other genitourinary diseases were excluded from the controls. Every case was matched with two controls by age within ±2 years.

### 2.3. Ethical Approval

The study was approved by the Medical Ethics Committee of Wuhan University of Science and Technology. Written informed consent was obtained from the study subjects who were assured of confidentiality by the use of anonymous questionnaires. Verbal consent was also sought from community leaders prior to the focus group discussions.

### 2.4. Data Collection

An anonymous questionnaire was designed by experts on health statistics and urology, and all of the collectors were trained before the questionnaire survey. According to the results of a presurvey, the questionnaire and plan were modified. The questionnaire was proved to be valid. Finally, the questionnaire consisted of five major domains of items, including demographics (age, degree of education, occupation, medical insurance, and average monthly income), lifestyle (frequency of eating fast food, time of using a mobile per day, smoking, drinking, drinking tea, and sedentariness), working situation (occupational hazards, night shift), psychological status (severity of bad mood, stress of work, economic stress of family, self-health cognition, and spousal relationship), and a physical exam. The item of occupational hazards determined by type of work was asked by questionnaire investigators. The questionnaire was self-administered after informed consent unless the participant was illiterate.

### 2.5. Statistical Analysis

The collected questionnaires were collated manually for the first time. They were then checked again while the data were entered into the database set up by Epi database. Data were analyzed using descriptive statistical methods, the chi-square test, and conditional logistic regression analysis. The Statistical Package for Sciences (SPSS) software version 17.0 (SPSS Inc., Chicago, IL, USA) was used for data analysis. The significance level was set at *P* < 0.05.

## 3. Results

### 3.1. Sociodemographic Characteristics

A total of 279 patients and 558 controls were recruited to participate in this retrospective survey. All of the participants completed the questionnaire. The age of all of the subjects ranged from 24 to 59 years. The mean age of all of the subjects was 43.30 ± 7.92 years. The mean ages of patients and controls were 43.57 ± 8.09 years and 43.16 ± 7.84 years, respectively.

For cases, 72.04% of subjects were aged between 30 and 49 years. Only one subject was illiterate, and most (82.79%) had a middle school or high school diploma. A total of 78.14% of the patients were employed as skilled laborers. A total of 86.73% of the patients had medical insurance for urban workers except for 3 who were reported as self-paying when seeing a doctor. More than 80% of the patients had an average monthly income less than 3000 CNY (483 USD), and only 10 patients had an average monthly salary of more than 5000 CNY (805 USD) (Table 1).

Similarly, 72.22% of the controls were aged between 30 and 49 years. Most of the controls were employed as skilled laborers. A total of 86.73% of the controls had medical insurance for urban workers, whereas five subjects were reported as self-paying when seeing a doctor. Almost 80% of the control subjects' average monthly income was less than 3000 CNY (483 USD). All of the controls received school education, and 82.79% had a secondary school or high school diploma (Table 1).

### 3.2. Chi-Square Test for Single-Factor Analysis

Table 2 shows the values of significant risk factors.  Table 3 shows the results of single-factor analysis. Several factors including “time of using a mobile per day,” “smoking,” “drinking,” “drinking tea,” and “sedentariness” did not have significant difference between patients and controls. The odds of CP/CPPS for subjects who were exposed to chemical factors in occupational workplace increased by approximately 104% (95% confidence interval [CI], 1.416–2.941). A night shift caused the odds of CP/CPPS to increase by approximately 53% for workers. The odds of CP/CPPS increased with the frequency of eating fast food (odds ratio, 1.32; 95% CI, 1.022–1.703). The time of using a mobile phone per day also affected the odds of CP/CPPS with positive correlation (odds ratio, 1.152; 95% CI, 1.027–1.293), which means, when spending more time on mobile phone, there will be more risk on suffering CP/CPPS. The risk of CP/CPPS increased with the degree of mood (e.g., sadness, anxiety, and depression), stress of work, economic stress of family, the level of self-health cognition, and spousal relationship, with odds ratios of 1.691 (95% CI, 1.350–2.119), 1.323 (95% CI, 1.124–1.557), 1.167 (95% CI, 1.006–1.354), 1.384 (95% CI, 1.170–1.637), and 1.513 (95% CI, 1.147–1.996), respectively.

### 3.3. Conditional Logistic Regression for Multiple-Factor Analysis

Nine significant factors selected by the chi-square test were used to build the regression model. Occupational hazards were set as classification variables, using the forward Wald method. Probability values for stepwise entry and removal were 0.05 and 0.10, respectively. Finally, four factors were included in the regression model (Table 4). Chemical factors, night shift, degree of mood (e.g., sadness, anxiety, and depression), and poor self-health cognition increased the odds of CP/CPPS by 93%, 46%, 62%, and 30%, respectively.

## 4. Discussion

Prostatitis has become increasingly more common, and age is not a limiting factor. Given the complexity of prostatitis, a systematic classification was provided by the NIH, including category I (acute bacterial prostatitis), category II (chronic bacterial prostatitis), category III (chronic bacterial prostatitis/CPPS), and category IV (asymptomatic inflammatory prostatitis) [16]. Among these four categories, chronic bacterial prostatitis/CPPS has become a recognized intractable disease. In China, a previous study indicated that most urological surgeons considered chronic bacterial prostatitis/CPPS as a clinical syndrome, and different treatment protocols were used to relieve pain, improve voiding symptoms, and improve quality of life [17]. Treatment protocols for bacterial prostatitis/CPPS that are used by urological surgeons include drug therapy (95%), changing lifestyle (88.9%), and psychotherapy (79.9%). Drugs include botanical drugs (84.5%), adrenergic alpha-antagonists (79%), and antibiotics (64%) [17]. Based on the results of a review of Medline articles, many individual therapies, including antibiotics, anti-inflammatory medications, neuromodulators, alpha blockers, pelvic floor physical therapy, and cognitive behavior therapy, have been evaluated in the treatment of CP/CPPS. Each therapy has been found to have varying efficiency in alleviating symptoms [18]. In a clinical study, the effect of combination therapy was analyzed in a single specialized prostatitis clinic; the result showed that a clinically appreciable reduction of ≥6 points of the total NIH-CPSI score was achieved in 77.5% of patients subjected to combination therapy for a period of 6 months [19]. Multimodal therapy that includes pharmacotherapy, baths, prostate massage, and pelvic floor physical therapy may help patients to control the symptoms of CP/CPPS [11]. Another study in China showed that 65% of CP patients undergo long-term routine treatment 12 times per year, and most CP patients are not satisfied with the effectiveness of the costly treatment [13].

In addition, the quality of life obviously declines in patients with CP/CPPS. Wenninger et al. [20] evaluated the effect of chronic nonbacterial prostatitis on the quality of life and functional status. They found that the mean Sickness Impact Profile score in men with chronic nonbacterial prostatitis was 7.5, which was greater than that for the general population. Additionally, the most severe effect of CP/CPPS appeared to be on social interaction in their study [20].

Thus, many epidemiologic studies have been done to find key risk factors of the disease and help people change their lifestyle to reduce the risk of CP/CPPS. Lan et al. [21] carried out a multicenter case-control study between June 2005 and May 2008 in China. They showed that urinary system infection, frequent masturbation, a cold climate, prostatomegaly, mental stress, high altitude, little exercise, and alcohol addiction might be risk factors of CP/CPPS [21]. This study also found that severity of mood (e.g., sadness, anxiety, and depression) might be related to CP/CPPS. Zhao et al. [22] conducted a retrospective case-control study of clinical data from 322 CP/CPPS patients (case group) and 341 non-CP/CPPS patients (control group). They showed an association between foreskin length and the odds of CP/CPPS. When the foreskin length covered up more than half of the glans penis, there were greater odds for CP/CPPS [22]. A literature review performed by Pontari and Ruggieri showed that the symptoms of CP/CPPS appeared to result from interplay between psychological factors and dysfunction in the immune, neurological, and endocrine systems [23]. Another study performed in northwest China suggested that oxidative stress and cytokines might be involved in the pathological process and aggravation of symptoms [24]. These results suggested that further experimental study, like cellular and molecular level research, should be done. This study could not explain whether exposure to cold was a risk factor because patients and controls came from the same region. A multinational observational study indicated that factors of the severity of symptoms of CP/CPPS varied between regions [25]. This previous study showed that effects of exposure to cold (*P* = 0.1856) and abdominal symptoms (*P* = 0.1119) were highest in Finland, those of education level (*P* = 0.0151), sexual activity (*P* = 0.0574), and erectile dysfunction (*P* = 0.0151) were highest in Germany/Switzerland, and those of age (*P* = 0.0698) were highest in Italy [25].

Dietary habit is often considered to have a considerable effect on CP/CPPS. According to our chi-square test results, among the lifestyle factors in the questionnaire, only “frequency of eating fast food” was significant. Finally, this factor was not included in the regression model. Many foods, such as spicy food, coffee, alcoholic beverages, and tea, can exacerbate the symptoms of patients with CP/CPPS, while others, such as docusate, psyllium, water, herbal teas, and polycarbophil, can ameliorate symptoms [26]. Another case-control study showed that the risk factors of CP include spicy food and drinking alcohol [27]. In our study, the degree of mood (e.g., sadness, anxiety, and depression) was significant in single-factor analysis and multiple-factor analysis. Many previous studies obtained similar results that depression might be involved in the development and clinical course of CP/CPPS [28–32]. In fact, depression and CP/CPPS may share, at least in part, several common pathophysiological mechanisms [7, 33]. It was demonstrated that the prostate gland responds to emotional stimulation through the autonomic nervous system; an experimental evidence also supports the theory that psychological stress may contribute to dysfunction of the prostate [34]. However, other studies have suggested that CP patients experience an increased risk of depressive disorders compared with non-CP patients [30], which meant psychological problems occurred after the disease. Our study also showed that night shifts might increase the risk of suffering from CP/CPPS by approximately 46%. When working at night, workers needed to overcome much more difficulties, such as fatigue, sleepiness, loneliness, and inattention. It was reported that staying up late was a risk factor of CP/CPPS [27]. In our study, although chemical factors had an effect on CP/CPPS; this factor needs to be studied further because the chemical substances were unknown. Some chemical substances and/or their metabolites might have a negative effect on the prostate when they penetrate the human body's protective barrier and enter the body. But this needs further studies because occupational hazards were only based on job duties without fast-field analysis, and the chemical substances were unknown. Self-health cognition was also a significant factor, but there might have been bias. Diseases, especially those that can reduce the quality of life, can change one's self-health cognition to a great extent. Therefore, despite the statistical significance of self-health cognition, it had no practical significance.

There were some limitations in our study. First, because this study was a retrospective study, it could not provide sufficient evidence of a causal relationship between risk factors and CP/CPPS. Second, the questionnaire was self-designed, despite its reliability, and bias might have been present. Third, chemical occupational factors have not been divided into particular toxicity or hazard, which may be confusing.

## 5. Conclusions

Many studies have shown a relationship between CP/CPPS and potential risk factors. An increasing number of researchers support the viewpoint that CP/CPPS is a clinical syndrome with an unclear or unknown pathogenesis. Our study shows that chemical factors, night shifts, the moods of sadness, anxiety, depression, and poor self-health cognition may affect CP/CPPS. Although there are many limitations in this study, our results might provide instructive information for patients and urologists.

## Figures and Tables

**Table 1:** Sociodemographic characteristics of respondents (*n* = 837).

Characteristic	Value	Cases *n* (%)	Controls *n* (%)
Age	24~	15 (5.38%)	42 (7.53%)
	30~	70 (25.09%)	136 (24.37%)
	40~	131 (46.95%)	267 (47.85%)
	50~	63 (22.58%)	113 (20.25%)

Degree of education	No formal education	1 (0.36%)	0 (0.00%)
	Primary school	2 (0.72%)	19 (3.40%)
	Junior high school	105 (37.63%)	179 (32.08%)
	Senior high or technical secondary school	126 (45.16%)	258 (46.24%)
	Junior college or above	45 (16.13%)	102 (18.28%)

Occupation	Unskilled; for example, trader, farming	20 (7.17%)	34 (6.09%)
	Skilled labor; for example, driver, blue-collar worker	218 (78.14%)	450 (80.65%)
	Professional; for example, teacher, healthcare worker, office worker	6 (2.15%)	20 (3.58%)
	Others	35 (12.54%)	54 (9.68%)

Medical insurance	Medical insurance for urban workers	242 (86.73%)	484 (86.73%)
	Rural cooperative medical service	30 (10.75%)	57 (10.21%)
	Commercial insurance	3 (1.08%)	6 (1.08%)
	Self-paying	3 (1.08%)	5 (0.90%)
	Others	1 (0.36%)	6 (1.08%)

Average monthly income	Less than 1000 CNY	40 (14.34%)	71 (12.72%)
	1000–2000 CNY	106 (37.99%)	206 (36.92%)
	2000–3000 CNY	78 (27.96%)	161 (28.85%)
	3000–4000 CNY	31 (11.11%)	62 (11.11%)
	4000–5000 CNY	14 (5.02%)	30 (5.38%)
	More than 5000 CNY	10 (3.58%)	28 (5.02%)

**Table 2:** Values of significant risk factors.

Variable	Value		
CP/CPPS	No = 0	Yes = 1	

Occupational hazards	No factors = 0	Physical factors = 1	Chemical factors = 2
	Biological factors = 3	Other factors = 4	

Night shift	Yes = 1	No = 2	

Frequency of eating fast food	Frequent = 1	Once in a while = 2	Never = 3

Time of using a mobile per day	Less than half an hour = 1	Half an hour to one hour = 2	
	One hour to two hours = 3	Two hours to three hours = 4	
	Three hours to four hours = 5	More than four hours = 6	

Severity of mood (e.g., sadness, anxiety, depression)	Not a bit = 1	A bit = 2	Medium = 3
	Very serious = 4	Extremely serious = 5	

Stress of work	Not a bit = 1	A bit = 2	Medium = 3
	Very serious = 4	Extremely serious = 5	

Economic stress of family	Not a bit = 1	A bit = 2	Medium = 3
	Very serious = 4	Extremely serious = 5	

Self-health cognition	Beyond comparison = 1	Very good = 2	Good = 3
	Common = 4	Bad = 5	

Spousal relationship	Very good = 1	Common = 2	Bad = 3

CP/CPPS: chronic prostatitis/chronic pelvic pain syndrome.

**Table 3:** Results of the chi-square test for single-factor analysis.

Factors	*B*	SE	Wald	df	Sig.	Exp (*B*)	95.0% CI for Exp (*B*)	
No occupational hazards	—	—	15.372	4	0.004	—	—	—
Chemical factors	0.713	0.187	14.620		**0.000**	**2.040**	1.416	2.941
Not on night shift	−0.426^a^	0.144	8.769	1	**0.003**	**0.653**	0.493	0.866
Low frequency of eating fast food	−0.278^a^	0.130	4.540	1	**0.033**	**0.758**	0.587	0.978
Time of using a mobile per day	0.142	0.059	5.793	1	**0.016**	**1.152**	1.027	1.293
Severity of mood (e.g., sadness, anxiety, depression)	0.525	0.115	20.848	1	**0.000**	**1.691**	1.350	2.119
Stress of work	0.280	0.083	11.317	1	**0.001**	**1.323**	1.124	1.557
Economic stress of family	0.155	0.076	4.147	1	**0.042**	**1.167**	1.006	1.354
Self-health cognition	0.325	0.086	14.355	1	**0.000**	**1.384**	1.170	1.637
Spousal relationship	0.414	0.141	8.593	1	**0.003**	**1.513**	1.147	1.996

^a^Two negative values were obtained because the variables “not on night shifts” and “low frequency of eating fast food” were two protective factors of CP/CPPS.

**Table 4:** Results of conditional logistic regression for multiple-factor analysis.

Factors	*B*	SE	Wald	df	Sig.	Exp (*B*)	95.0% CI for Exp (*B*)	
No occupational hazards	—	—	11.919	4	0.018	—	—	—
Physical factors	0.269	0.255	1.117	1	0.291	1.309	0.794	2.158
Chemical factors	0.657	0.193	11.546	1	**0.001**	**1.929**	1.321	2.819
Biological factors	0.417	0.651	0.411	1	0.522	1.518	0.424	5.438
Others	0.360	0.250	2.074	1	0.150	1.433	0.878	2.339
Not on night shift	−0.376^a^	0.149	6.365	1	**0.012**	**0.687**	0.513	0.920
Severity of mood (e.g., sadness, anxiety, depression)	0.482	0.120	16.213	1	**0.000**	**1.619**	1.280	2.046
Self-health cognition	0.265	0.090	8.741	1	**0.003**	**1.304**	1.094	1.555

^a^A negative value was obtained because the variable “not on night shifts” was a protective factor of CP/CPPS.
